# Association between changes in handgrip strength and depression in Korean adults: a longitudinal panel study

**DOI:** 10.1038/s41598-022-18089-9

**Published:** 2022-08-11

**Authors:** Hyunkyu Kim, Wonjeong Jeong, Seung Hoon Kim, Yu Shin Park, Sung-In Jang, Eun-Cheol Park

**Affiliations:** 1 Department of Preventive Medicine, Yonsei University College of Medicine, 50 Yonsei-ro, Seodaemun-gu, Seoul, 03722 Republic of Korea; 2 Institute of Health Services Research, Yonsei University, Seoul, Republic of Korea; 3 Department of Psychiatry, Yonsei University College of Medicine, Seoul, Republic of Korea; 4 Department of Public Health, Graduate School, Yonsei University, Seoul, Republic of Korea; 5 Department of Preventive Medicine, Gachon University College of Medicine, Incheon, Republic of Korea

**Keywords:** Psychiatric disorders, Risk factors

## Abstract

Depression in older adults is a global socioeconomic burden. Identifying factors, such as physical activity or exercise that can help prevent depression is important. We aimed to investigate the relationship between changes in handgrip strength and the presence of depression using longitudinal, nationwide data of older Korean adults. Data from the Korean Longitudinal Study of Aging were used in this study. A total of 6783 participants who had undergone a handgrip strength test and completed the short-form Center for Epidemiologic Studies Depression Scale (CESD-10-D) questionnaire from 2006 to 2018 were included. General estimating equations were used to assess the temporal effect of the changes in handgrip strength on depression. A decrease in handgrip strength was associated with high CESD-10-D scores (β = 0.1889 in men, β = 0.1552 in women). As a continuous variable, handgrip strength was negatively correlated with CESD-10-D scores(β = − 0.0166 in men, β = − 0.0196 in women). Changes in the handgrip strength were associated with depressive symptoms in our longitudinal study. Those who experienced a decrease in handgrip strength had severe depressive symptoms compared to those with unchanged or increased handgrip strength. These findings can be used to guide general health policies for the prevention of depression.

## Introduction

Globally, depression is a major health problem and a leading cause of socioeconomic burden due to its growing prevalence and associated high suicide rate^1–5^. In particular, South Korea has been struggling with a higher prevalence of suicidality than other countries of the Organization for Economic Co-operation and Development and thus has focused on mitigating the associated risks for the last few decades^6,7^. To address this problem, previous studies in South Korea have mainly focused on biological and psychological treatment methods for depression, such as medications and cognitive or behavioral psychotherapy^8–10^. However, it is important to find methods that involve lifestyle changes, such as eating habits, nutrition, exercise, and sleep pattern, to reduce the prevalence of depression. Among studies on the methods for modifying lifestyle, a previous study suggested that increased levels of exercise can help prevent depression^11^.

Patients with depression exhibit various symptoms, including reduced physical activity, with some patients showing an extreme form of inactivity because of catatonia. Researchers and clinicians have focused on the symptom-reducing function of physical activity or exercise among depressive patients. In fact, previous studies showed that exercise led to a reduction in depressive symptoms and that cognitive behavior therapy combined with exercise led to favorable outcomes^12–15^. These studies prompted many countries, including South Korea, to include exercise in their depression treatment guidelines^16,17^.

While the therapeutic potential of exercise in alleviating depression has been established, its efficacy as a depression prevention measure remains unclear. Previous studies found an association between initial physical activity and reduced depression^18^ and between handgrip strength asymmetry and depression^19^. However, very few studies have investigated the association between exercise and the prevalence of depression using a longitudinal study design. Moreover, previous studies did not use methods that allowed for instant measurement of the exercise status of the participants, thus limiting the utilization of the results in clinical settings. Therefore, this study used handgrip strength, which can be measured instantly and easily, as a proxy for the muscle power status of a participant^20^. Further, changes in handgrip strength can also be measured regularly to determine the exercise status of participants in a given period^21–23^. This study aimed to investigate the relationship between changes in handgrip strength and depressive symptoms in a Korean adult population cohort selected from a panel study.

## Methods

### Study population and data

The data analyzed in this study were extracted from the Korean Longitudinal Study of Aging database (KLoSA). The KLoSA is a longitudinal panel survey of national representative samples of community-dwelling adults aged above 45 years and has been conducted every two years since 2006^24^. The baseline data were gathered in 2006, where 10,254 Korean adults were interviewed by trained interviewers. The survey collected information on family background, demographics, family, health, employment status, income, and assets and included questionnaires on subjective expectations and subjective quality of life. In 2018, the seventh wave was conducted, and the effective sample number was 6,136 from the original panels and 804 from the newly included panels. In this study, we used biannual survey data from 2006 to 2018, resulting in seven rounds of data. After removing data with missing values for the study variables, 6,793 participants (3,052 men and 3,731 women) were included in this study. The baseline characteristics of the included and excluded individuals are shown in Table S1. For statistical analysis, each change in handgrip strength from 2006 to 2018, rather than each participant, was treated as an individual case.

### Measures

The short-form Center for Epidemiologic Studies Depression Scale (CESD-10-D) was used for measuring depressive symptoms. The validity of the Korean version of CESD-10-D for screening of depression is well established^25,26^. The participants were asked to answer 10 questions about their depressive condition using a binomial scoring system. The KLoSA provides a raw score by adding the scores of all the answers, and this score ranges from 0 to 10, with high scores indicating high severity of depression. We used a CESD-10-D cut-off score of 3 to determine the association between the change in handgrip strength and the presence of depressive symptoms .

### Handgrip strength

Handgrip strength was measured in kilograms using a handgrip dynamometer (Hand Grip Meter 6103, Tanita, Tokyo, Japan). The participants were asked to squeeze the dynamometer twice with each hand, and the highest value among the four trials was used in this study. The participants who refused to perform the test due to physical problems were excluded from the data analysis. To analyze the relationship between the change in handgrip strength and the presence of depressive symptoms, the changes in both domains over the previous year were recorded. The continuous variable of handgrip strength was categorized into two groups: (1) decreased and (2) same or increased; the analyses were conducted assuming the presence of continuous changes in the recorded values across the two groups.

### Covariates

Demographic and health-related factors were included as covariates in the analysis. The following demographic characteristics were included: age, educational level, dwelling region, working status, household income, participation in social activities, and the number of cohabiting generations^27^. The following health-related factors were included: smoking/alcohol use status, number of chronic medical conditions, body mass index (BMI), and perceived health status. All the covariates were measured using survey questionnaires. The KLoSA provides the number of chronic medical conditions for each participant, including hypertension, diabetes, cancers, chronic lung diseases, liver disease, cardiac disease, cerebrovascular diseases, psychiatric disease, arthritis, prostate disease, dementia, and other chronic diseases. All multivariable models were controlled for all of the covariates unless stated otherwise.

### Statistical analysis

All statistical analyses were performed separately for men and women to rule out the effect of sex on depression. Analysis of variance was used to compare the general characteristics of the groups^27^. A generalized estimating equation (GEE) model was used for regression analysis of CESD-10 scores, change in handgrip strength, and other covariates. CESD-10 score was included as the outcome variable, and other variables in Table 1 were included in the GEE model. We used normal distribution with the identity link function for continuous variables and binomial distribution and logit link function for binary outcome variables. The temporal variable was the wave, i.e., every 2 years, and person ID was used to identify repeated subjects using the unstructured working correlation matrix for the GEE model. The analysis was conducted twice: first, after dividing the change in handgrip strength into two groups, and second, after setting the change in handgrip strength as a continuous variable. The results are presented as regression coefficients (β) and standard errors.

**Table 1:** Baseline characteristics of the study population according to the short-form Center for Epidemiologic Studies Depression Scale (CESD-10-D) scores.

	Men (n = 3052)					Women (n = 3731)				
	Participants		CESD-10-D			Participants		CESD-10-D		
	N	%	Mean	SD	p-value	N	%	Mean	SD	p-value
**Changes in handgrip strength**					0.8602					0.7542
Same or Increased	1213	39.7	3.042	2.756		1691	45.3	3.793	2.883	
Decreased	1839	60.3	3.058	2.698		2040	54.7	3.820	2.891	
**Age, years**					< 0.0001					< 0.0001
45–54	845	27.7	2.512	2.445		1150	30.8	2.925	2.569	
55–64	954	31.3	2.698	2.585		1083	29.0	3.547	2.849	
65–74	890	29.2	3.430	2.826		1002	26.9	4.515	2.898	
≥ 75	363	11.9	4.309	2.884		496	13.3	4.994	2.897	
**Education level**					< 0.0001					< 0.0001
Elementary school or less	924	30.3	3.868	2.913		2018	54.1	4.466	2.930	
Middle school	529	17.3	3.149	2.805		642	17.2	3.402	2.735	
High school	1085	35.6	2.690	2.568		897	24.0	2.858	2.566	
University or beyond	514	16.8	2.247	2.146		174	4.7	2.557	2.427	
**Region**					< 0.0001					0.0003
Metropolitan	1266	41.5	2.694	2.539		1589	42.6	3.497	2.821	
Small or medium cities	1009	33.1	3.105	2.804		1226	32.9	3.879	2.962	
Rural	777	25.5	3.566	2.812		916	24.6	4.250	2.838	
**Working status**					< 0.0001					< 0.0001
Working	1937	63.5	2.590	2.519		1230	33.0	3.221	2.660	
Non-working	1115	36.5	3.854	2.870		2501	67.0	4.096	2.951	
**Household income**					< 0.0001					< 0.0001
Quartile 1 (low)	609	20.0	4.118	2.866		988	26.5	4.968	2.947	
Quartile 2	825	27.0	3.238	2.775		997	26.7	3.876	2.839	
Quartile 3	865	28.3	2.690	2.576		910	24.4	3.313	2.737	
Quartile 4 (high)	753	24.7	2.401	2.409		836	22.4	2.894	2.549	
**Participation in social activities**					< 0.0001					< 0.0001
No	538	17.6	4.126	3.002		819	22.0	4.722	2.988	
Yes	2514	82.4	2.822	2.600		2912	78.0	3.550	2.805	
**Smoking**					0.0776					< 0.0001
Current	1188	38.9	3.115	2.748		109	2.9	5.459	3.114	
Former	789	25.9	3.118	2.673		38	1.0	4.605	3.150	
Never	1075	35.2	2.933	2.724		3584	96.1	3.749	2.862	
**Alcohol intake**					0.4527					0.4280
Yes	1926	63.1	2.911	2.659		715	19.2	3.418	2.774	
No	1126	36.9	3.292	2.809		3016	80.8	3.900	2.906	
**Number of chronic medical conditions**					< 0.0001					0.5450
None	175	51.6	2.639	2.558		1697	45.5	3.11	2.68	
1	905	29.7	3.190	2.737		1143	30.6	3.90	2.90	
≥ 2	572	18.7	3.970	2.884		891	23.9	5.00	2.86	
**Number of cohabiting generations**					0.4463					0.0791
Couple	1424	46.7	3.270	2.786		1790	48.0	4.085	2.949	
Two generation	1287	42.2	2.810	2.602		1436	38.5	3.498	2.812	
Over two generation	341	11.2	3.053	2.814		505	13.5	3.705	2.782	
**BMI**					0.0005					0.0494
Underweight	92	3.0	4.000	3.045		113	3.0	4.301	3.062	
Normal weight	1341	43.9	3.293	2.787		1647	44.1	3.760	2.878	
Overweight	1027	33.7	2.888	2.627		1039	27.8	3.774	2.831	
Obesity	559	18.3	2.615	2.579		820	22.0	3.883	2.913	
Severe obesity	33	1.1	3.091	2.788		112	3.0	3.768	3.148	
**Perceived health status**					< 0.0001					< 0.0001
Healthy	1787	58.6	2.357	2.383		1707	45.8	2.755	2.527	
Average	865	28.3	3.527	2.747		1231	33.0	4.017	2.716	
Unhealthy	400	13.1	5.128	2.809		793	21.3	5.749	2.795	

Underweight: BMI < 18.5; normal weight: 18.5 ≤ BMI < 23; overweight: 23 ≤ BMI < 25; obesity: 25 ≤ BMI < 30; severe obesity: 30 ≤ BMI.

*BMI* body mass index, *SD* standard deviation, *CESD-10-D* Shorter form of the Center for Epidemiologic Studies Depression Scale.

Subgroup analyses were performed to assess the interaction between handgrip strength change and other variables that were associated with the CESD-10 score further. We conducted subgroup analyses for age, working status, participation in social activities, number of chronic medical conditions, and perceived health status. All analyses were carried out using SAS software version 9.4 (SAS Institute, Cary, North Carolina, USA), and the results were considered statistically significant if the p-value was < 0.05 and very highly significant if the p-value was < 0.001.

### Ethical considerations

The KLoSA study was approved by Statistics Korea of the Korean Government (Approval number: 33602 and the Institutional Review Board of Korea National Institute for Ethics Policy (P01-201909-22-002). The survey was conducted after acquiring written informed consent of the participants by the trained study interviewer of KLoSA survey. This study was approved as exempt by the Institutional Review Board of Yonsei University’s Health System (4-2021-0307). This study adhered to the principles of the Declaration of Helsinki.

## Results

The baseline characteristics of the study population, stratified by sex, are presented in Table 1. A total of 6,793 participants (3,052 men and 3,731 women) were included in the analysis. Of the participants, 39.7% of the men and 45.3% of the women showed either no changes in handgrip in the first two waves or an increase in the second wave. In the unadjusted analysis, we found no significant difference in CESD-10-D scores between the two handgrip strength groups in both sexes. However, the other covariates such as age, educational level, region, working status, household income, participation in social activities, number of chronic medical conditions, BMI, and perceived health status significantly differed in CESD-10-D scores for both sexes.

Table 2 shows the multiple regression analysis results for the CESD-10-D score and groups of change in handgrip after adjusting for the covariates. Compared to the same or increased handgrip strength group, the decreased handgrip strength group showed regression coefficients of 0.1889 in men and 0.1552 in women, which were highly significant at p < 0.0001. A decrease in handgrip strength was associated with an increase in CESD-10-D scores in both sexes. The results of the other covariates are shown in Table 2. Table 3 shows the results of multiple regression analysis between CESD-10-D score and handgrip strength change as continuous values using the same covariates from Table 2. The regression coefficients were − 0.0166 in men and − 0.0196 in women, with a high significance level of p < 0.0001. These results suggest that the change in handgrip strength was negatively associated with the CESD-10-D total score in both sexes. Table 4 shows that the decreased handgrip strength group showed a higher odds ratio (OR) for depression (OR = 1.18, 95% confidence interval [CI] 1.10–1.27 in men, OR = 1.09, 95% CI 1.02–1.16 in women) after dividing the participants into two groups using the CESD-10-D cut-off score of 3.

**Table 2:** Results of the generalized estimating equation analysis of handgrip strength change in the two groups and short-form Center for Epidemiologic Studies Depression Scale scores.

	Men			Women		
	β	S.E.	p-value	β	S.E.	p-value
**Changes in handgrip strength**						
Same or increased	Ref			Ref		
Decreased	0.1889	0.0417	< 0.0001	0.1552	0.0390	< 0.0001
**Age, years**						
45–54	Ref			Ref		
55–64	− 0.1467	0.0704	0.0372	− 0.1234	0.0656	0.0598
65–74	− 0.1658	0.0939	0.0774	− 0.1689	0.0893	0.0586
≥ 75	− 0.0880	0.1174	0.4535	− 0.1139	0.1089	0.2955
**Education level**						
Elementary school or less	0.7297	0.1101	< 0.0001	0.7245	0.1454	< 0.0001
Middle school	0.4702	0.1148	< 0.0001	0.3180	0.1492	0.0331
High school	0.3426	0.0920	0.0002	0.0770	0.1386	0.5788
University or beyond	Ref			Ref		
**Region**						
Metropolitan	Ref			Ref		
Small or medium cities	0.2786	0.0794	0.0005	0.3028	0.0722	< 0.0001
Rural	0.1342	0.0850	0.1143	0.1490	0.0782	0.0568
**Working status**						
Working	Ref			Ref		
Non-working	0.5319	0.0668	< 0.0001	0.3145	0.0580	< 0.0001
**Household income**						
Quartile 1 (low)	0.2382	0.0998	0.0171	0.5562	0.0873	< 0.0001
Quartile 2	0.1686	0.0817	0.0390	0.3516	0.0766	< 0.0001
Quartile 3	0.0526	0.0682	0.4405	0.1007	0.0698	0.1490
Quartile 4 (high)	Ref			Ref		
**Participation in social activities**						
No	0.4939	0.0746	< 0.0001	0.2227	0.0632	0.0004
Yes	Ref			Ref		
**Smoking**						
Current	0.0747	0.0821	0.3628	0.6841	0.1953	0.0005
Former	− 0.1596	0.0798	0.0456	0.4384	0.2427	0.0709
Never	Ref			Ref		
**Alcohol intake**						
Yes	− 0.0860	0.0639	0.1782	− 0.1416	0.0740	0.0557
No	Ref			Ref		
**Number of chronic medical conditions**						
None	Ref			Ref		
1	− 0.0047	0.0701	0.9461	0.1335	0.0689	0.0526
≥ 2	− 0.0410	0.0861	0.6341	0.3419	0.0813	< 0.0001
**Number of cohabiting generations**						
Couple	0.0141	0.1078	0.8961	− 0.1809	0.0944	0.0552
Two generation	0.1243	0.1033	0.2289	0.0291	0.0933	0.7548
Over two generation	Ref			Ref		
**BMI**						
Underweight	0.1099	0.1603	0.4932	0.1297	0.1492	0.3847
Normal weight	Ref			Ref		
Overweight	− 0.1384	0.0624	0.0265	− 0.0192	0.0584	0.7426
Obesity	− 0.2770	0.0787	0.0004	− 0.1326	0.0690	0.0547
Severe obesity	− 0.5115	0.2828	0.0704	− 0.2835	0.1855	0.1264
**Perceived health status**						
Healthy	Ref			Ref		
Average	0.3562	0.0546	< 0.0001	0.3485	0.0503	< 0.0001
Unhealthy	1.6343	0.0893	< 0.0001	1.5368	0.0744	< 0.0001

Underweight: BMI < 18.5; normal weight: 18.5 ≤ BMI < 23; overweight: 23 ≤ BMI < 25; obesity: 25 ≤ BMI < 30; severe obesity: 30 ≤ BMI.

*BMI* body mass index, *S.E.* standard error.

**Table 3:** Results of the generalized estimating equation analysis of handgrip strength change as a continuous variable and short-form Center for Epidemiologic Studies Depression Scale scores.

	Men			Women		
	β	S.E.	p-value	β	S.E.	p-value
Handgrip strength change (kg)	− 0.0166	0.0030	< 0.0001	− 0.0196	0.0039	< 0.0001

All covariates in Table 2 were included in this analysis.

*S.E.* standard error.

**Table 4:** Results of the GEE analysis of handgrip strength change and depression.

	Depression(3 ≤ CESD-10-D)			
	Men		Women	
	Adjusted OR	95% CI	Adjusted OR	95% CI
**Changes in Handgrip strength**				
Same or increased	Ref		Ref	
Decreased	1.18	(1.10–1.27)	1.09	(1.02–1.16)

All variables in Table 2 were included in the GEE model.

The results of the subgroup analysis are shown in Table S2. When we grouped the data by age, all age groups showed high CESD-10 scores when their handgrip strength had decreased, except the oldest male and female groups, which showed no significant relationship between handgrip change and depression.

## Discussions

We found that a decrease in handgrip strength during the previous 2 years was associated with depressive symptoms in Korean adults. The participants whose handgrip strength decreased had reported higher CESD-10-D scores than those whose handgrip strength had remained the same or increased in the same period. Furthermore, we found that handgrip strength was negatively correlated with the CESD-10-D score in our study population.

The results of the present study are generally consistent with those of previous studies, namely that handgrip strength was associated with a high prevalence of depression or an increase in depressive symptoms. For example, one study found that weak handgrip strength was associated with a high odds ratio for depression in low- and middle-income countries. Although it was a cross-sectional study, the odds ratio of depression was 1.45 in the weak hand strength group, suggesting that handgrip strength was directly related to depressive symptomology^28^. Furthermore, a cross-sectional study involving community-dwelling adults in the USA also found that sarcopenia, denoted by decreased handgrip strength, was associated with the presence of depression^29^. Another cohort study conducted in Japan by Fukumori et al. reported that low baseline handgrip strength was associated with depressive symptoms^30^. Reduced handgrip strength was also associated with the development of depression after 1 year in a longitudinal analysis; this study suggested that a higher handgrip strength might prevent depression.

The mechanism underlying the association between handgrip strength and the presence of depressive symptoms has not yet been established. However, several hypotheses have suggested that exercise has antidepressant effects, giving us a clue about the association between muscle strength, a result of exercise, and depressive symptoms. One hypothesis is that exercise relieves depressive symptoms by reversing depression-induced atrophy of brain structures. Atrophy of brain structures such as the hippocampus, anterior cingulate cortex, and prefrontal cortex has been reported in patients with depression^31^. Importantly, several studies have shown that exercise increases the volume of the hippocampus, anterior accumbens, and prefrontal cortex, thus providing antidepressant effects^32–34^. Activated muscle or increased muscle mass might produce antidepressant effects by elevating the level of serotonin in the brain. In fact, since muscles consume branched-chain amino acids as their metabolism substrates, the serum concentration of single amino acids such as tryptophan increases. High levels of tryptophan in the serum and cerebrospinal fluid lead to increased levels of serotonin and dopamine, resulting in a reduction in depression^35,36^. Exercise has also been found to increase the levels of brain-derived neurotrophic factor (BDNF), which is known to regenerate as well as enhance the function of the hippocampus. Given that decreased levels of BDNF have been found to be related to depression in older patients, exercise may not only reverse but also prevent depressive symptoms by increasing BDNF levels^37^. These previous studies suggest that exercise might prevent depression, and since we can measure the exercise status via handgrip strength, it might be associated with depression in individuals.

This study had some limitations. First, we used survey-based data and excluded non-responders; thus, the results might have been affected by this bias. Individuals with severe depression or severe sarcopenia might not have accurately responded to the survey or may not have been included; thus, our results might have been underestimated. Second, we could not determine the biological risk factors for depression since we used only a survey-based database. As several biological factors have been established as risk factors for depression in adults, future studies should analyze them in regression models^38^. Third, causation could not be determined because of the lack of a prospective design. We used the change in handgrip strength between two waves and analyzed its association with depressive symptoms in the subsequent wave to minimize the risk of reciprocal causation. However, depressive symptoms in the previous waves could have caused a decrease in handgrip strength. Thus, future studies using a prospective design including interventions of strengthening exercises are needed to establish the causal relationship between changes in handgrip strength and depressive symptomology.

Despite these limitations, our study has many strengths. We conducted the analysis with a relatively large sample size that represented the general adult population of South Korea and used a longitudinal design. These results can be applied to the general Korean population to establish health care policies or conduct future studies. Second, we used standardized tools to measure muscle strength. Handgrip strength is easily measurable and can be standardized across different groups and studies. Third, we used the change in handgrip strength rather than baseline strength; thus, the results could be analyzed as the exercise or physical activity status of the previous period. Finally, our results suggest that increasing handgrip strength by modifying lifestyle habits is a useful strategy for preventing depressive symptoms in adults.

In conclusion, this longitudinal, large-sized study showed that change in handgrip strength was associated with depressive symptoms in South Korea. A decrease in handgrip strength was associated with severe depressive symptoms compared to no change or increased handgrip strength. Future studies exploring the underlying mechanisms of this association as well as the preventive effects of increasing handgrip strength on depressive symptoms may provide valuable strategies for treating and preventing depressive symptoms.

## Supplementary Information

Supplementary Tables.

## Data Availability

The datasets analyzed during the current study are available in the KLoSA repository, https://survey.keis.or.kr/eng/klosa/databoard/List.jsp.

## References

[1] Hu T-W (2004). The economic burden of depression and reimbursement policy in the Asia Pacific region. Australas. Psychiatry.

[2] Johnston KM, Powell LC, Anderson IM, Szabo S, Cline S (2019). The burden of treatment-resistant depression: A systematic review of the economic and quality of life literature. J. Affect. Disord..

[3] Judd, L. L., Paulus, M. P., Wells, K. & Rapaport, M. Socioeconomic burden of subsyndromal depressive symptoms and major depression in a sample of the general population. (1996). 10.1176/ajp.153.11.1411 8890673

[4] Sussman M, O’sullivan AK, Shah A, Olfson M, Menzin J (2019). Economic burden of treatment-resistant depression on the US Health Care System. J. Manag. Care Spec. Pharm..

[5] Tanner J-A (2020). Economic burden of depression and associated resource use in Manitoba Canada. Can. J. Psychiatry.

[6] Lee S-U (2018). Changing trends in suicide rates in South Korea from 1993 to 2016: a descriptive study. BMJ Open.

[7] Myung W (2015). Paraquat prohibition and change in the suicide rate and methods in South Korea. PLoS ONE.

[8] Kok RM, Reynolds CF (2017). Management of depression in older adults: a review. JAMA.

[9] McHugh, R. K., Whitton, S. W., Peckham, A. D., Welge, J. A. & Otto, M. W. Patient preference for psychological vs. pharmacological treatment of psychiatric disorders: A meta-analytic review. *J. Clin. Psychiatry* **74**, 595 (2013). 10.4088/JCP.12r07757 PMC4156137 23842011

[10] Oakes P, Loukas M, Oskouian RJ, Tubbs RS (2017). The neuroanatomy of depression: A review. Clin. Anat..

[11] Mammen G, Faulkner G (2013). Physical activity and the prevention of depression: A systematic review of prospective studies. Am. J. Prev. Med..

[12] Abdollahi A (2017). Effect of exercise augmentation of cognitive behavioural therapy for the treatment of suicidal ideation and depression. J. Affect. Disord..

[13] Euteneuer F (2017). Immunological effects of behavioral activation with exercise in major depression: An exploratory randomized controlled trial. Transl. Psychiatry.

[14] Kvam S, Kleppe CL, Nordhus IH, Hovland A (2016). Exercise as a treatment for depression: A meta-analysis. J. Affect. Disord..

[15] Kim H (2020). Sex differences in type of exercise associated with depression in South Korean adults. Sci. Rep..

[16] Park S-C (2014). Evidence-based, non-pharmacological treatment guideline for depression in Korea. J. Korean Med. Sci..

[17] Ranjbar, E. *et al.* Depression and exercise: a clinical review and management guideline. *Asian J. Sports Med.* **6** (2015). 10.5812/asjsm.6(2)2015.24055 PMC4592762 26448838

[18] Harvey SB (2018). Exercise and the prevention of depression: results of the HUNT cohort study. Am. J. Psychiatry.

[19] Hurh K, Park Y, Kim GR, Jang S-I, Park E-C (2021). Associations of handgrip strength and handgrip strength asymmetry with depression in the elderly in Korea: A cross-sectional study. J. Prev. Med. Public Health.

[20] Seong JY, Ahn HY, Park Y, Shin S, Ha I-H (2020). Association between aerobic exercise and handgrip strength in adults: A cross-sectional study based on data from the Korean National Health and Nutrition Examination Survey (2014–2017). J. Nutr. Health Aging.

[21] Haider S (2016). Associations between daily physical activity, handgrip strength, muscle mass, physical performance and quality of life in prefrail and frail community-dwelling older adults. Qual. Life Res..

[22] Shin HI (2017). Relation between respiratory muscle strength and skeletal muscle mass and hand grip strength in the healthy elderly. Ann. Rehabil. Med..

[23] Arokiasamy P, Selvamani Y, Jotheeswaran AT, Sadana R (2021). Socioeconomic differences in handgrip strength and its association with measures of intrinsic capacity among older adults in six middle-income countries. Sci. Rep..

[24] Jang, S.-N. Korean longitudinal study of ageing (KLoSA): Overview of research design and contents. 1–9. 10.1007/978-981-287-080-3_55-1 (2015).

[25] Cho, M. J. & Kim, K. H. Diagnostic validity of the CES-D(Korean Version) in the assessment of DSM-III-R major depression. *J. Korean Neuropsychiatr. Assoc.* **32**, 381–399 (1993).

[26] Shin, S. Y. Validity Study of Short Forms of the Korean version Center for Epidemiologic Studies Depression Scale(CES-D), Vol. Master of philosophy in medicine vii, 48 장 (Seoul National University, 2011).

[27] Kim H (2021). Association between change in handgrip strength and cognitive function in Korean adults: a longitudinal panel study. BMC Geriatr..

[28] Ashdown-Franks, G. *et al.* Handgrip strength and depression among 34,129 adults aged 50 years and older in six low- and middle-income countries. *J. Affect. Disord.* **243**, 448–454. 10.1016/j.jad.2018.09.036 (2019). 10.1016/j.jad.2018.09.036 30273883

[29] Brooks, J. M. *et al.* Depression and handgrip strength among U.S. adults aged 60 years and older from NHANES 2011–2014. *J. Nutr. Health Aging* **22**, 938–943. 10.1007/s12603-018-1041-5 (2018). 10.1007/s12603-018-1041-5 PMC6168750 30272097

[30] Fukumori N (2015). Association between hand-grip strength and depressive symptoms: Locomotive Syndrome and Health Outcomes in Aizu Cohort Study (LOHAS). Age Ageing.

[31] Gujral S, Aizenstein H, Reynolds CF, Butters MA, Erickson KI (2017). Exercise effects on depression: Possible neural mechanisms. Gen. Hosp. Psychiatry.

[32] Bugg JM, Head D (2011). Exercise moderates age-related atrophy of the medial temporal lobe. Neurobiol. Aging.

[33] Erickson KI (2009). Aerobic fitness is associated with hippocampal volume in elderly humans. Hippocampus.

[34] Gordon BA (2008). Neuroanatomical correlates of aging, cardiopulmonary fitness level, and education. Psychophysiology.

[35] Heijnen S, Hommel B, Kibele A, Colzato LS (2015). Neuromodulation of aerobic exercise—a review. Front. Psychol..

[36] Patrick RP, Ames BN (2015). Vitamin D and the omega-3 fatty acids control serotonin synthesis and action, part 2: Relevance for ADHD, bipolar disorder, schizophrenia, and impulsive behavior. FASEB J..

[37] Erickson KI, Miller DL, Roecklein KA (2012). The aging hippocampus: interactions between exercise, depression, and BDNF. Neuroscientist.

[38] Krishnan KRR (2002). Biological risk factors in late life depression. Biol. Psychiatry.

