# Supplementary material for: Association between changes in handgrip strength and depression in Korean adults: a longitudinal panel study
Source: Sci Rep. 2022 Aug 11;12:13643. doi: 10.1038/s41598-022-18089-9 (PMC9372156; doi:10.1038/s41598-022-18089-9)
Supplement: Supplementary file 1 — Supplementary Tables. [file 41598_2022_18089_MOESM1_ESM.docx]

| **Table S1. Baseline Characteristics of included and excluded population** | | | |  |  |
| --- | --- | --- | --- | --- | --- |
| **Variables** | **Included participants** | | **Excluded participants** | | **P value** |
|  |  |  |  |  |  |
|  | **N** | **%** | **N** | **%** |  |
| **Total** | **6,783** | **100** | **1,905** | **100** |  |
| **Changes in Handgrip strength** |  |  |  |  | <0.0001 |
| Same or Increased | 2,904 | 42.8 | 276 | 14.5 |  |
| Decreased | 3,879 | 57.2 | 985 | 51.7 |  |
| Missing |  |  | 644 | 33.8 |  |
| **CESD-10 Score** |  |  |  |  | <0.0001 |
| Mean(SD) | 3.4675 | 2.8384 | 4.8672 | 3.1179 |  |
| Missing |  |  | 52 | 2.7 |  |
| **Sex** |  |  |  |  | <0.0001 |
| Men | 3052 | 45.0 | 714 | 37.5 |  |
| Women | 3731 | 55.0 | 1191 | 62.5 |  |
| Missing |  |  | 0 | 0.0 |  |
| **Age** |  |  |  |  | <0.0001 |
| 45-54 | 1995 | 29.4 | 255 | 13.4 |  |
| 55-64 | 2037 | 30.0 | 364 | 19.1 |  |
| 65-74 | 1892 | 27.9 | 612 | 32.1 |  |
| ≥75 | 859 | 12.7 | 674 | 35.4 |  |
| Missing |  |  | 0 | 0.0 |  |
| **Education level** |  |  |  |  | <0.0001 |
| Elementary school or less | 2942 | 43.4 | 1188 | 62.4 |  |
| Middle school | 1171 | 17.3 | 230 | 12.1 |  |
| High school | 1982 | 29.2 | 328 | 17.2 |  |
| University or beyond | 688 | 10.1 | 156 | 8.2 |  |
| Missing |  |  | 3 | 0.2 |  |
| **Region** |  |  |  |  |  |
| Metropolitan | 2855 | 42.1 | 909 | 47.7 | <0.0001 |
| Small or Medium Cities | 2235 | 33.0 | 550 | 28.9 |  |
| Rural | 1693 | 25.0 | 446 | 23.4 |  |
| Missing |  |  | 0 | 0.0 |  |
| **Working status** |  |  |  |  |  |
| Working | 3167 | 46.7 | 429 | 22.5 | <0.0001 |
| Non-working | 3616 | 53.3 | 1476 | 77.5 |  |
| Missing |  |  | 0 | 0.0 |  |
| **Household income** |  |  |  |  | <0.0001 |
| Quartile 1 (low) | 1579 | 23.3 | 715 | 37.5 |  |
| Quartile 2 | 1840 | 27.1 | 572 | 30.0 |  |
| Quartile 3 | 1775 | 26.2 | 340 | 17.8 |  |
| Quartile 4 (high) | 1589 | 23.4 | 278 | 14.6 |  |
| Missing |  |  | 0 | 0.0 |  |
| **Participation in social activities** |  |  |  |  | <0.0001 |
| No | 1357 | 20.0 | 732 | 38.4 |  |
| Yes | 5426 | 80.0 | 1173 | 61.6 |  |
| Missing |  |  | 0 | 0.0 |  |
| **Smoking** |  |  |  |  | <0.0001 |
| Current | 4659 | 68.7 | 1385 | 72.7 |  |
| Former | 827 | 12.2 | 235 | 12.3 |  |
| Never | 1297 | 19.1 | 285 | 15.0 |  |
| Missing |  |  | 0 | 0.0 |  |
| **Alcohol Intake** |  |  |  |  | <0.0001 |
| Yes | 2641 | 38.9 | 519 | 27.2 |  |
| No | 4142 | 61.1 | 1386 | 72.8 |  |
| Missing |  |  | 0 | 0.0 |  |
| **Number of chronic medical conditions** |  |  |  |  | <0.0001 |
| None | 3272 | 48.2 | 615 | 32.3 |  |
| 1 | 2048 | 30.2 | 590 | 31.0 |  |
| ≥2 | 1463 | 21.6 | 700 | 36.7 |  |
| Missing |  |  | 0 | 0.0 |  |
| **Number of cohabiting generation** |  |  |  |  |  |
| Couple | 3214 | 47.4 | 910 | 47.8 | <0.0001 |
| Two generation | 2723 | 40.1 | 664 | 34.9 |  |
| Over two generation | 846 | 12.5 | 331 | 17.4 |  |
| Missing |  |  | 0 | 0.0 |  |
| **BMI** |  |  |  |  |  |
| Underweight | 145 | 2.1 | 86 | 4.5 | <0.0001 |
| Normal weight | 1379 | 20.3 | 325 | 17.1 |  |
| Overweight | 2066 | 30.5 | 454 | 23.8 |  |
| Obesity | 2988 | 44.1 | 794 | 41.7 |  |
| Severe obesity | 205 | 3.0 | 157 | 8.2 |  |
| Missing |  |  | 89 | 4.7 |  |
| **Perceived health status** |  |  |  |  | <0.0001 |
| Healthy | 3494 | 51.5 | 484 | 25.4 |  |
| Average | 2096 | 30.9 | 590 | 31.0 |  |
| Unhealthy | 1193 | 17.6 | 831 | 43.6 |  |
| Missing |  |  | 0 | 0.0 |  |

| **Table S2. Subgroup analysis of the association between change in handgrip strength and CESD-10** | | | | | | |  |
| --- | --- | --- | --- | --- | --- | --- | --- |
|  |  |  | **Change in Handgrip strength** | | | | **p for Interaction** |
|  |  |  | **Same or Increased** | **Decreased** | | |  |
|  |  |  | **β** | **β** | **S.E** | **p-Value** |  |
| **Men** | **Age** | 45-54 | Ref. | 0.1164 | 0.1013 | 0.2508 | 0.5444 |
|  |  | 55-64 | Ref. | 0.2695 | 0.0733 | 0.0002 |  |
|  |  | 65-74 | Ref. | 0.2614 | 0.0798 | 0.0011 |  |
|  |  | ≥75 | Ref. | 0.1334 | 0.1044 | 0.2015 |  |
|  | **Working status** | Working | Ref. | 0.2014 | 0.0526 | 0.0001 | 0.9323 |
|  |  | Non-working | Ref. | 0.1810 | 0.0701 | 0.0099 |  |
|  | **Participation in social activities** | No | Ref. | 0.1068 | 0.1080 | 0.3229 | 0.3059 |
|  |  | Yes | Ref. | 0.2176 | 0.0680 | 0.0014 |  |
|  | **Number of chronic medical conditions** | None | Ref. | 0.2275 | 0.0603 | 0.0002 | 0.4958 |
|  |  | 1 | Ref. | 0.1769 | 0.0839 | 0.0349 |  |
|  |  | ≥2 | Ref. | 0.1617 | 0.0842 | 0.0547 |  |
|  | **Perceived health status** | Healthy | Ref. | 0.2297 | 0.0547 | <0.0001 | 0.5850 |
|  |  | Average | Ref. | 0.0875 | 0.0785 | 0.2651 |  |
|  |  | Unhealthy | Ref. | 0.3287 | 0.1377 | 0.0169 |  |
| **Women** | **Age** | 45-54 | Ref. | 0.2200 | 0.0827 | 0.0078 | 0.0103 |
|  |  | 55-64 | Ref. | 0.2691 | 0.0708 | 0.0001 |  |
|  |  | 65-74 | Ref. | 0.1879 | 0.0758 | 0.0131 |  |
|  |  | ≥75 | Ref. | -0.0454 | 0.0929 | 0.6253 |  |
|  | **Working status** | Working | Ref. | 0.1817 | 0.0678 | 0.0074 | 0.6635 |
|  |  | Non-working | Ref. | 0.1536 | 0.0487 | 0.0016 |  |
|  | **Participation in social activities** | No | Ref. | 0.1030 | 0.0888 | 0.2462 | 0.6943 |
|  |  | Yes | Ref. | 0.1768 | 0.0479 | 0.0002 |  |
|  | **Number of chronic medical conditions** | None | Ref. | 0.1802 | 0.0617 | 0.0035 | 0.7182 |
|  |  | 1 | Ref. | 0.1745 | 0.0729 | 0.0167 |  |
|  |  | ≥2 | Ref. | 0.1530 | 0.0731 | 0.0365 |  |
|  | **Perceived health status** | Healthy | Ref. | 0.1836 | 0.0593 | 0.0020 | 0.7069 |
|  |  | Average | Ref. | 0.0738 | 0.0704 | 0.2943 |  |
|  |  | Unhealthy | Ref. | 0.2470 | 0.0995 | 0.0130 |  |
